# Microwave-Assisted Rapid Synthesis of Cu_2_S/CoO Heterostructured Composites for High-Performance Asymmetric Supercapacitors

**DOI:** 10.3390/molecules31203715

**Published:** 2026-10-16

**Authors:** Yunxia Zhao, Yu Yang, Qianwen Duan, Shuxin Gong, Bowen Du, Zhen Lu, Feng Feng

**Affiliations:** School of Chemistry and Chemical Engineering, Shanxi Datong University, Datong 037009, China

**Keywords:** microwave synthesis, Cu_2_S/CoO heterostructured composites, asymmetric supercapacitors

## Abstract

Transition metal sulfides and oxides are attractive for supercapacitor electrodes due to rich redox sites and high theoretical capacitances. Yet single-phase materials suffer from poor structural stability and slow charge transfer, restricting practical applications. Herein, a facile microwave route is developed to prepare Cu_2_S/CoO composite electrodes. Experimental results demonstrate that the heterogeneous interface between Cu_2_S and CoO induces a prominent synergistic effect, which greatly boosts the redox activity of the electrode. The optimized Cu_2_S/CoO composite exhibits a specific capacitance of 2898 F g^−1^ at 1 A g^−1^, and maintains 84.1% of its initial capacitance after 30,000 continuous cycles, revealing remarkable cycling durability. The asymmetric supercapacitor fabricated with the Cu_2_S/CoO negative electrode delivers an energy density of 186.45 Wh kg^−1^ at a power density of 800.02 W kg^−1^, and this performance outperforms most recently reported analogous electrode systems. This research offers a practical route for the fast fabrication of high-performance bimetallic composite electrodes through microwave treatment, and supplies useful references for the structural engineering and performance optimization of advanced electrochemical energy storage materials.

## 1. Introduction

Escalating global energy shortages, together with the widespread adoption of portable electronics, are driving the exploration of reliable electrochemical energy storage systems, which stands as a key research focus within materials science. The rational scheduling of low-carbon energy systems has put forward higher requirements for high-performance and high-reliability energy storage components [1,2]. Featuring prominent power density, favorable cycling durability and rapid charge–discharge response, supercapacitors exhibit promising application prospects in hybrid electric vehicles, smart grids, and wearable electronics [3,4,5,6,7]. Serving as the central component of supercapacitors, electrode materials dictate the overall device performance via their microstructure, electrical conductivity, and electrochemical reactivity. For this reason, rationally designing and synthesizing electrode materials with a large specific capacitance, favorable rate performance, and durable cycling life is highly demanded to promote further advances of supercapacitors [8,9].

Transition metal sulfides (TMSs) and transition metal oxides (TMOs) [10,11,12] are considered promising alternatives to noble-metal-based electrode materials owing to high theoretical specific capacitance, abundant resources, and environmental friendliness. Among them, Cu_2_S, a typical p-type semiconductor, possesses a unique layered crystal structure, high intrinsic electrical conductivity, and abundant redox-active sites, which has attracted considerable attention in the field of supercapacitors in recent years [13,14]. For instance, Samdhyan et al. [15] prepared CuS electrodes via a hydrothermal method, which exhibited a specific capacitance of 232.5 F g^−1^ at 1 A g^−1^ with a capacitance retention of 72.6% after 500 cycles. Gao et al. [16] fabricated Cu_2_S/Cu electrodes via an impregnation method, achieving a high capacitance of 1219 F g^−1^ at 5 mA cm^−2^ and retaining 92.4% of its initial capacitance after 5000 cycles. Xu et al. [17] synthesized nanorod materials grown by in situ etching on copper foam, which delivered a specific capacity of 448.8 C g^−1^ at 5 mA cm^−2^ and retained 89% of its initial capacitance after 10,000 cycles. Despite these encouraging achievements, pristine Cu_2_S electrodes still suffer from serious volume expansion and structural collapse upon long-term cycling, so their cycling stability cannot meet the requirements for practical applications.

On the other hand, CoO, a typical transition metal oxide, possesses an ultrahigh theoretical specific capacitance (approximately 3000 F g^−1^) and favorable electrochemical reversibility [18,19]. Nevertheless, its low intrinsic electrical conductivity greatly restricts the rate capability and long-term cycling stability [20,21]. To overcome the performance drawbacks of single-component materials, constructing binary or multicomponent composites with synergistic effects has been demonstrated as an effective strategy [22,23,24,25,26,27]. For example, Lu et al. [28] fabricated hierarchical tubular CoO/Co–Cu–S heterostructures assembled from nanoneedles through a multi-step route including calcination treatment. The optimized sample delivered a high specific capacitance of 2300 F g^−1^ at 2.0 A g^−1^, with a capacitance retention of roughly 95% after 5000 cycles.

Accordingly, the integration of highly conductive Cu_2_S and high-capacity CoO is expected to achieve synergistic performance enhancement. On the one hand, Cu_2_S acts as a rapid electron transport pathway to optimize the charge collection efficiency between CoO particles. On the other hand, CoO with a high theoretical capacitance endows the composite electrode with higher energy density. More importantly, lattice strain and electronic reconstruction generated at the biphase heterointerfaces may expose abundant electrochemical active sites, accelerating ion diffusion and faradaic reaction kinetics [29,30]. Recent studies on transition metal-based composite electrodes have verified the effectiveness of such a structural design strategy. Pang et al. [31] synthesized CuS/Co_3_S_4_ composites via a hydrothermal treatment, which exhibited a specific capacitance of 1483 F g^−1^ at 1 A g^−1^ and retained 1038.1 F g^−1^ at 20 A g^−1^. Khan et al. [32] prepared CuS/FeSe_2_ nanocomposites with a specific capacitance of 821.3 F g^−1^ at 1 A g^−1^, significantly superior to pristine CuS (248.3 F g^−1^) and FeSe_2_ (508 F g^−1^), along with a capacitance retention of 80.4%. These findings clearly demonstrate that the construction of multicomponent heterostructures is a valid and universal strategy to enhance the electrochemical performance of transition metal compound electrode materials.

However, most previously reported strategies for composite electrodes rely on multi-step complex processes such as hydrothermal treatment and high-temperature calcination. Such methods are time-consuming, energy-intensive, and exhibit poor batch reproducibility, which greatly limits their scalable practical applications. Benefiting from uniform thermal distribution, ultrafast reaction kinetics, facile operation, and high energy utilization efficiency, microwave synthesis technology has emerged as a versatile and advanced technique for the rapid fabrication of inorganic functional materials. Accordingly, this work develops a facile, one-step, and ultrafast microwave strategy to prepare spherical Cu_2_S/CoO heterostructured composites. This method realizes homogeneous biphase integration and tight interfacial bonding within a short reaction time. Systematic characterizations were implemented to analyze the phase constituents, morphological features, and electrochemical properties of the prepared composites. Electrochemical measurements demonstrate that the optimized Cu_2_S/CoO electrode delivers an ultrahigh specific capacitance of 2898 F g^−1^ at 1 A g^−1^ and maintains 84.1% of its initial capacitance after 30,000 long-term cycles. Furthermore, the assembled asymmetric supercapacitor achieves a prominent energy density of 186.45 Wh kg^−1^ at a favorable power density. This work develops an economical and facile synthetic route to rapidly produce high-performance composite electrodes based on transition metal sulfide/oxide, offering meaningful insights into the structural modulation and performance optimization of advanced supercapacitor electrode materials.

## 2. Results and Discussion

### 2.1. Morphology and Structural Analysis of Cu_2_S/CoO Composite

**Figure 1:**
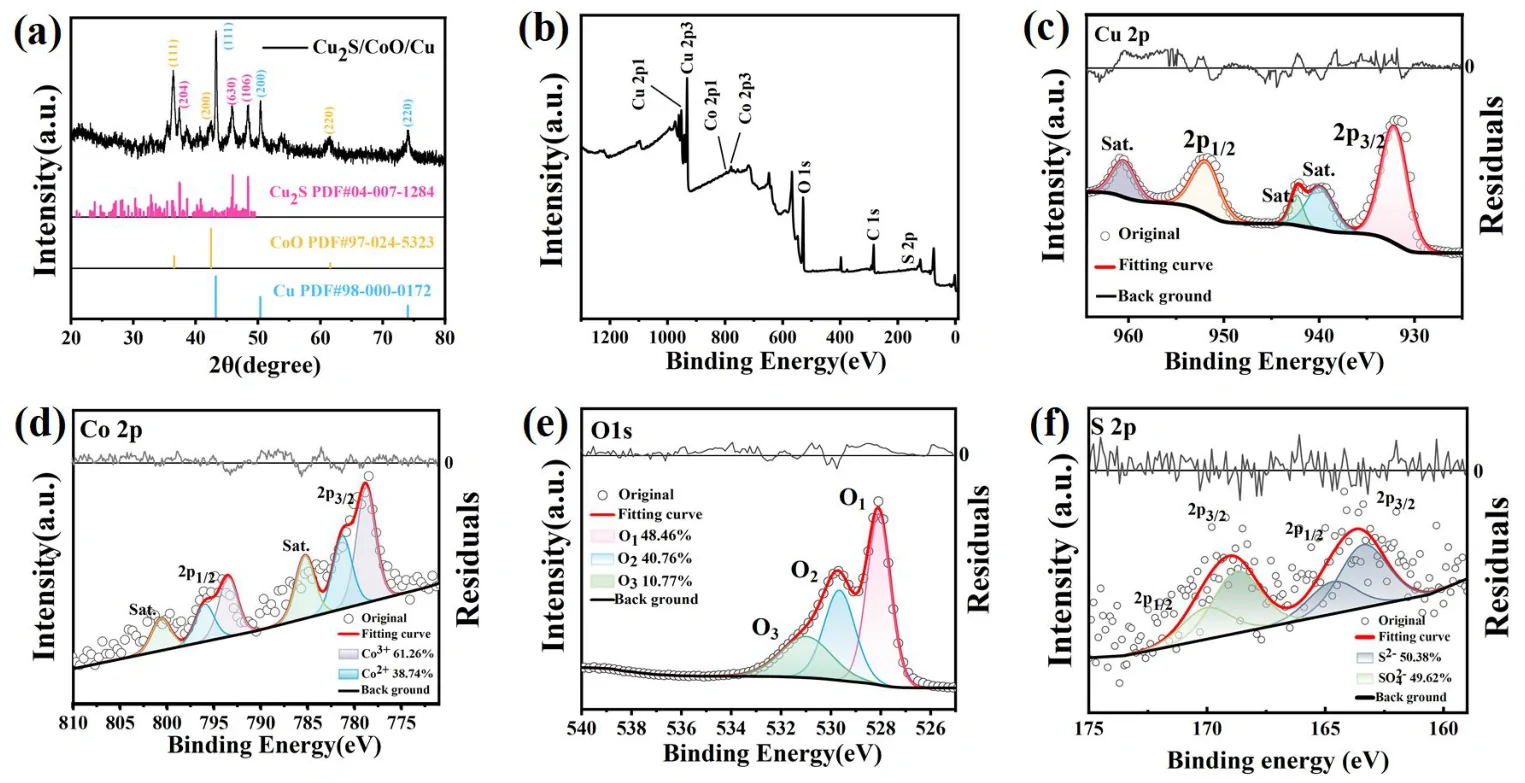
(**a**) XRD pattern of the Cu_2_S/CoO electrode material. (**b**–**f**) XPS spectra of the Cu_2_S/CoO electrode material.

X-ray diffraction (XRD) was used to investigate the crystal structure of the as-fabricated composite electrode, and the corresponding XRD pattern is shown in Figure 1a. The diffraction peaks located at 37.5°, 45.9°, and 48.5° can be indexed to the (204), (630), and (106) planes of Cu_2_S (PDF#04-007-1284), respectively. The peaks at 36.5°, 42.4°, and 61.5° correspond to the (111), (200), and (220) planes of CoO (PDF#97-024-5323). The diffraction signals at 43.3°, 50.4°, and 74.1° are assigned to the (111), (200), and (220) planes of the copper substrate (PDF#98-000-0172). The XRD results confirm the successful synthesis of the Cu_2_S/CoO composite electrode. X-ray photoelectron spectroscopy (XPS) was carried out to identify the surface elemental composition and chemical states of the sample. The characteristic photoelectron peaks of Cu, Co, S, and O were detected in the XPS survey spectrum (Figure 1b). Analysis of the valence states of metallic elements reveals the coexistence of multiple valence states for copper and cobalt within the composite, which provides the electrode with abundant redox-active sites. Cu mainly exists in the Cu^+^ state (Figure 1c). The peaks at 932.14 eV and 951.89 eV [33] are assigned to the Cu–S covalent bonds in Cu_2_S, the spin-orbit splitting distance is approximately 19.8 eV, and the peak area ratio constraint of the two is the theoretical value of 2:1, corresponding to the dominant valence state of copper. Minor satellite peaks for Cu^2+^ can also be detected, which originate from slight surface oxidation during material preparation and storage and generate trace CuO; such minor oxidation exerts negligible influence on the structure and electrochemical performance of the electrode. In Figure 1d, two major peaks located at 781.30 eV and 795.99 eV are attributed to Co^2+^ 2p_3_/_2_ and Co^2+^ 2p_1_/_2_, respectively, with a spin-orbit splitting energy of approximately 15.0 eV. Moreover, distinct shake-up satellite peaks appear at 785.29 eV and 800.63 eV [34,35], which are typical signatures of high-spin Co^2+^ (3d^7^), further confirming the presence of divalent cobalt. The peaks at 778.79 eV and 793.48 eV can be indexed to the Co^3+^ 2p_3_/_2_ and Co^3+^ 2p_1_/_2_ orbitals, indicating the formation of a surface oxide layer on the bimetallic composite. High-resolution spectra of nonmetallic elements further reveal the interfacial defects and bonding characteristics of the composite. In Figure 1e, O_1_ (528.08 eV) is attributed to lattice Co–O bonds in cobalt oxide; O_2_ (529.66 eV) corresponds to surface hydroxyl groups and oxygen vacancy defects [36]; and O_3_ (531.01 eV) originates from adsorbed water on the sample surface [37]. The relative area proportions of O_1_, O_2_, and O_3_ were 48.46%, 40.76%, and 10.77%, respectively. The relatively high proportion of interfacial oxygen (O_2_) indicates that a heterogeneous interface has formed between Cu_2_S and CoO. S (sulfur) the 2p spectrum shows multiple sets of characteristic responses (Figure 1f). The main peak double peaks at 163.39eV (S 2p_3_/_2_) and 164.69 eV (S 2p_1_/_2_) belong to the metal sulfide S^2−^, corresponding to the Cu–S covalent bond within Cu_2_S, which is the main component in the sample. The secondary bipeaks of 168.74 eV (S 2p_3_/_2_) and 170.04 eV (S 2p_1_/_2_) belong to the sulfate produced by surface oxidation [38]. In summary, the XPS results confirm the successful fabrication of the Cu_2_S/CoO electrode material. Cu and Co offer abundant valence state variations and create continuous multi-electron transfer pathways, thereby boosting the electrochemical activity of the material. Cu and Co offer abundant valence variations. Such mixed valence states originate from electron transfer at the heterointerface between Cu_2_S and CoO, confirming the interfacial interaction. This electronic modulation accelerates charge transfer during redox reactions and thereby improves the electrochemical activity of the material. The FTIR results (Figure S1) further confirm the coexistence of Co–O and Cu–S bonds and the interfacial electronic interaction of the Cu_2_S/CoO heterostructure.

**Figure 2:**
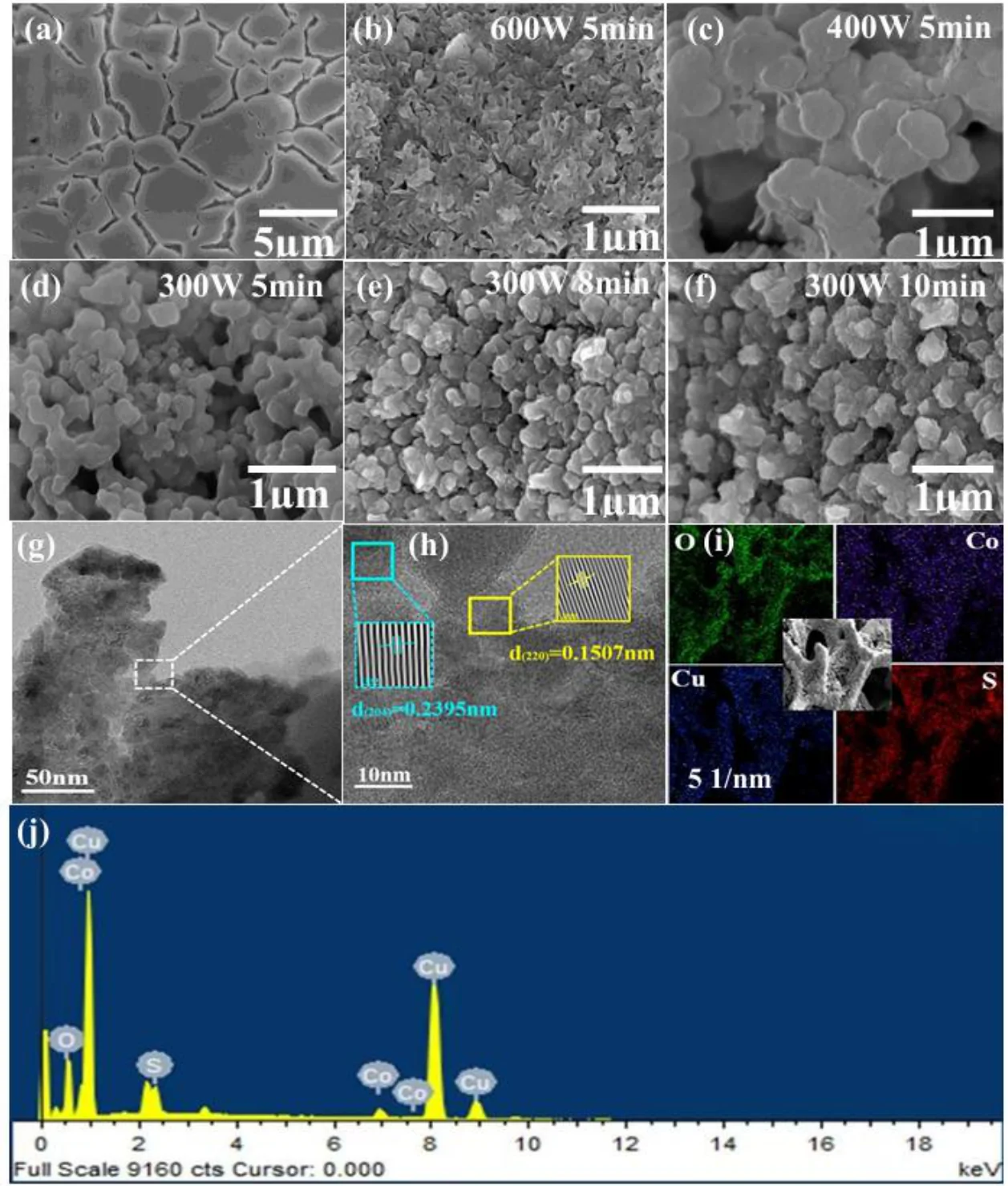
SEM and TEM images of the Cu_2_S/CoO electrode material. (**a**) Pure copper foam substrate. (**b**–**f**) Products synthesized under microwave conditions of 600 W for 5 min, 400 W for 5 min, 300 W for 5 min, 300 W for 8 min, and 300 W for 10 min, respectively. (**g**,**h**) HRTEM images (The scale bar of both insets is 1 nm). (**i**) Mapping. (**j**) EDS spectrum.

Scanning electron microscopy was utilized to explore the influence of microwave reaction parameters on the nanostructural morphology of the Cu_2_S/CoO composites. Pure copper foam was used as the control substrate (Figure 2a), which exhibited smooth bulk metallic skeletons without any nanoscale coating. The nucleation, growth, and agglomeration behavior of the composites was investigated by adjusting microwave power and reaction duration. Excessively high microwave power causes severe particle agglomeration and structural collapse. In contrast, moderate power coupled with an appropriate reaction time enables the growth of uniform and fine nanoparticles. Under high-power and short- duration conditions (600 W, 5 min and 400 W, 5 min; Figure 2b,c), a rapid temperature increase generates an extremely high instantaneous nucleation density. The resultant particles are densely stacked and severely aggregated into large clusters, which block most surface pores. Dense aggregation within these materials restricts the availability of electrochemically active sites, thus impairing their energy storage performance.

When the microwave power was reduced to 300 W, the nucleation rate decreased correspondingly, achieving a refined particle size. For the sample prepared at 300 W for 5 min (Figure 2d), the insufficient reaction time led to incomplete precursor conversion, uneven particle size distribution, and numerous unreacted amorphous clusters. By extending the reaction time to 8 min (Figure 2e), adequate crystal growth was realized, producing uniformly dispersed quasi-spherical nanoparticles with a consistent particle size of approximately 0.408 μm (Figure S2a). The abundant interconnected voids between nanoparticles effectively facilitate electrolyte infiltration and rapid charge transport. By further prolonging the reaction time to 10 min (Figure 2f), particle ripening and agglomeration took place with broadened particle-size distribution (~0.433 μm) (Figure S2b), and partial collapse of the porous structure was observed. Overall, the particle size gradually increases and the size distribution becomes broader with the extension of microwave reaction time.

Accordingly, the optimal microwave parameter was determined to be 300 W for 8 min. Under this condition, uniform, well-dispersed, and porous Cu_2_S/CoO nanostructures were successfully grown on copper foam. This favorable microstructure provides abundant active sites and continuous fast ion-transport pathways, laying a solid structural foundation for superior electrochemical performance. TEM (Figure 2g) and SEM (Figure 2e, for the optimal sample) images of the optimal Cu_2_S/CoO sample demonstrate that the composite is composed of numerous nanoparticles. The magnified microscopic view revealed nanograins with sizes ranging from 10–20 nm; these can also be seen from the TEM histogram (Figure S3). Two distinct sets of distinct lattice fringes with interplanar spacings of 0.1507 nm and 0.2395 nm were observed in the HRTEM image (Figure 2h), corresponding to the (220) crystal plane of CoO and the (204) crystal plane of Cu_2_S, consistent with the diffraction peaks in the XRD pattern (Figure 1a). The two lattice domains are closely adjacent without obvious interfacial gaps, confirming the formation of intimate heterointerfaces rather than simple physical mixing of two individual phases. The energy-dispersive X-ray spectrum (Figure 2i,j) of the optimal sample revealed Cu, Co, O, and S elements, further confirming the successful fabrication of the Cu_2_S/CoO composite electrode. The nitrogen adsorption–desorption characterization also verified the porous characteristics of this heterostructure (Figure S4).

### 2.2. Electrochemical Analysis of Cu_2_S/CoO Composites

**Figure 3:**
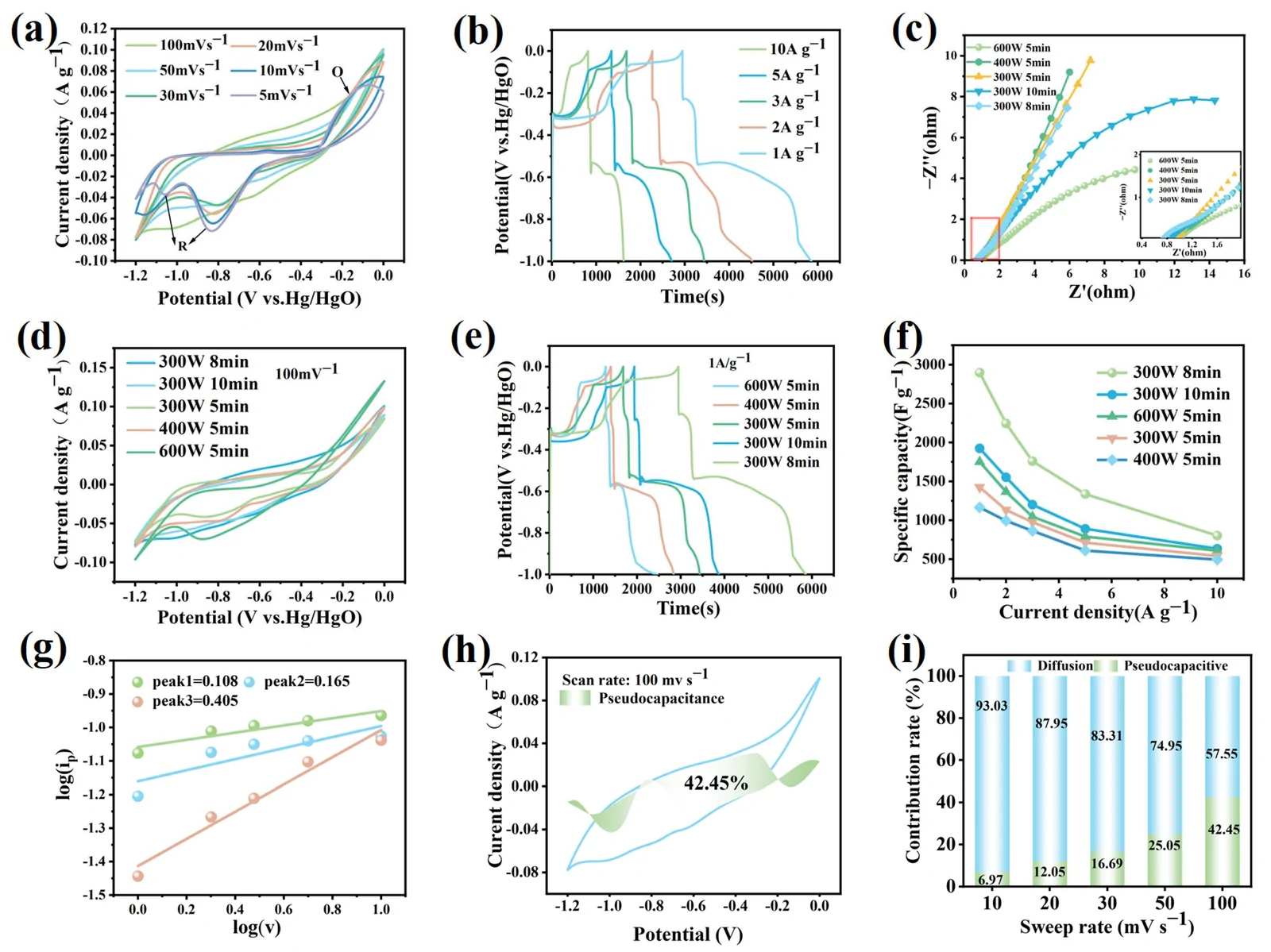
(**a**,**b**) CV and GCD curves of Cu_2_S/CoO obtained under optimal conditions (300 W 8 min). (**c**) EIS plots of samples prepared with different reaction parameters. (**d**–**f**) CV curves, GCD curves and specific capacitance under various microwave conditions. (**g**) Relationship between peak current and scan rate. (**h**) Capacitance fraction. (**i**) Relative contributions of charge storage.

Figure 3a presents the CV curves of the optimal Cu_2_S/CoO electrode synthesized at 300 W for 8 min, measured at various scan rates (5, 10, 20, 30, 50, and 100 mV s^−1^) in a three-electrode system. The GCD profiles illustrated in Figure 3b maintain good consistency with the redox peaks recorded in the CV measurements within the same voltage window. According to electrochemical computations, the optimized Cu_2_S/CoO electrode reaches 2898 F g^−1^ specific capacitance at 1 A g^−1^. To further understand how Cu_2_S/CoO heterostructures form throughout microwave synthesis, potential reaction routes are outlined as follows:

(NH_2_)_2_CS + H_2_O → CO_2_ + 2NH_3_ + H_2_S (1)

2Cu(CF) + H_2_S → Cu_2_S↓ + H_2_↑ (2)

Co^2+^ + 2OH^−^ → Co(OH)_2_↓ (NH_3_ + H_2_O ⇌ NH_4_^+^ + OH^−^) (3)

Co(OH)_2_↓ → CoO (4)

EIS characterizations at open-circuit potential from 10^−2^ Hz to 10^5^ Hz were performed to evaluate the charge and ion migration behaviors of the obtained electrodes (Figure 3c). On the Nyquist plot, the intercept at high frequencies on real axis denotes the Rs of the electrolyte. The semicircle size mirrors Rct, and the low-frequency slope links to Zw arising from ion diffusion [39,40,41]. Distinct impedance differences can be observed for samples prepared under different conditions. The Randles-type equivalent circuit diagram adopted for impedance fitting is displayed in Figure S5a. The series resistance of the device is 0.672 Ω, and the charge transfer resistance is 0.995 Ω. The Rct value suggests a small charge-transfer barrier at the electrode–electrolyte interface and fast interfacial reaction kinetics, confirming that the electrode can achieve efficient interfacial charge transfer during charge and discharge, which is beneficial to enhancing the rate performance of the device. The EIS fitting residuals (Figure S5b) distributed randomly near zero, demonstrating good fitting quality. The fitting parameters are detailed in Table S2. As the reaction time extends from 5 min to 10 min, the overall impedance continuously increases. The sample synthesized at 300 W for 10 min showed much higher impedance than those prepared at 300 W for 5 min and 8 min. This indicates that excessive reaction time elevates diffusion resistance and degrades the electrochemical performance. CV curves, GCD profiles, and specific capacitance values of electrodes fabricated under different conditions (Figure 3d–f) reveal that the sample treated at 300 W for 8 min possessed the largest enclosed area of CV curves and the highest anodic and cathodic peak current densities, delivering the optimum specific capacitance. With a fixed reaction time of 5 min, increasing the microwave power reduced both the CV integral area and peak current, which resulted in degraded capacitive behavior. At a constant microwave power of 300 W, the electrochemical performance first improved and then decayed upon prolonging reaction time, and 8 min was determined as the optimal parameter. Both insufficient and excessive reaction durations are unfavorable for energy storage performance. The correlation between peak current (I) and scan rate (v) obeys the kinetic equation as follows:

(5) $${I=a\cdot v}^{b}$$

The fitted slope b can be used to determine the rate-determining step. A value of b = 1 corresponds to surface-controlled faradaic processes, while b = 0.5 indicates diffusion-controlled behavior. The fitted b values of the redox peaks for this electrode are close to 0.5, demonstrating that its electrochemical reaction kinetics are mainly governed by ion diffusion (Figure 3g). CV contribution separation was performed to explore the charge storage mechanism (Figure 3h,i). As the scan rate increased, the diffusion-controlled contribution still remained as high as 57.55% at 100 mV s^−1^, confirming that diffusion-controlled battery-type behavior dominates over the entire tested scan rate range. The Cu_2_S/CoO heterointerface shortens the ion diffusion pathway and facilitates bulk ion penetration, supporting this diffusion-dominated charge storage property. The following equation was adopted to quantitatively analyze the charge storage contributions:

i(v) = k_1_ v + k_2_ v^1/2^ (6)

where k_1_v and k_2_v^1/2^ correspond to surface-controlled capacity and diffusion controlled capacity, respectively. The surface-controlled capacitance accounted for 42.45% of the total capacity of Cu_2_S/CoO, further confirming that the electrochemical process is dominated by ion diffusion.

**Figure 4:**
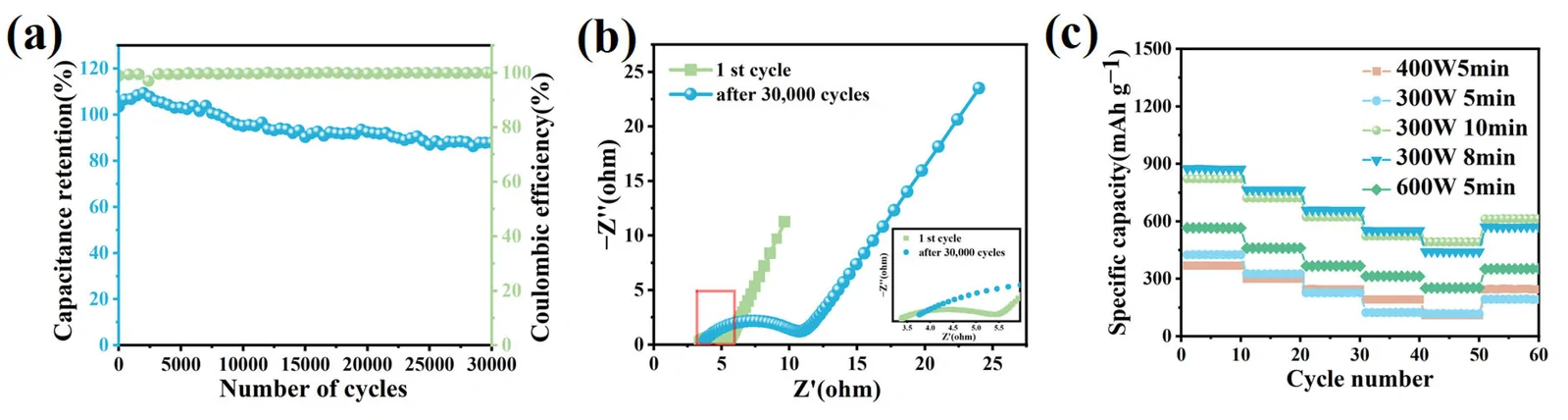
(**a**) Cycling stability and Coulombic efficiency. (**b**) Impedance variation before and after cycling. (**c**) Rate performance.

To evaluate the cycling stability of the electrode at low current density, a galvanostatic charge–discharge test was performed for 3000 cycles at 1 A g^−1^ (Figure S6). Using the initial specific capacitance at 1 A g^−1^ as the benchmark, the electrode delivered a capacitance retention of 89.3% after 3000 cycles, with an average Coulombic efficiency of 99% during the cycling process. Furthermore, a long-term cycling test was carried out at a high current density of 10 A g^−1^ for up to 30,000 cycles (Figure 4a). The capacitance retention was calculated based on the initial capacitance at 10 A g^−1^, and a retention of 84.1% was obtained after cycling, with the average Coulombic efficiency maintained at 99% throughout the test. These results demonstrate that the Cu_2_S/CoO electrode exhibits outstanding cycling stability under both low and high current densities. Electrochemical impedance spectroscopy (EIS) (Figure 4b) was measured before and after 30,000 galvanostatic charge–discharge (GCD) cycles to evaluate the evolution of interfacial resistance. The charge-transfer resistance increased from 0.99 Ω for the pristine electrode to approximately 2.04 Ω after long-term cycling. The moderate rise in Rct suggests mild deterioration of the electrode–electrolyte interface. Nevertheless, the electrode still maintains a capacitance retention of 84.1%, indicating that most electroactive sites remain available for surface-controlled charge storage even with increased interfacial resistance. Post-cycling SEM images (Figure S7) revealed obvious electrochemical reconstruction after long cycling. The initially aggregated nanoparticles evolved into uniform urchin-like microspheres assembled by dense nanospines. Fragmented debris could be observed on the surface of the reconstructed microspheres, originating from slight surface peeling of active materials during repeated redox reactions. No severe pulverization or bulk structural collapse was detected, confirming the favorable structural integrity of the Cu_2_S/CoO heterostructure. However, the formation of surface fragments and morphological rearrangement led to partial loss of electrical contact between active components, accompanied by slight irreversible phase transformation. The elevated Rct may stem from these morphological variations, together with minor interfacial side reactions and partial loss of accessible electroactive sites during continuous ion insertion/extraction, which accounts for the gradual capacitance decay. The rate capability measurements in Figure 4c reveal that the specific capacity of the electrode decreases stepwise with the increase in current density. A high specific capacity can be obtained at low current densities, while the capacity retention declines at elevated current densities, mainly originating from ion diffusion limitations and polarization effects under high current densities. Overall, the material delivers satisfactory capacity stability over a wide range of current densities. Table 1 summarizes the performance comparison between Cu_2_S/CoO and other reported electrode materials.

**Figure 5:**
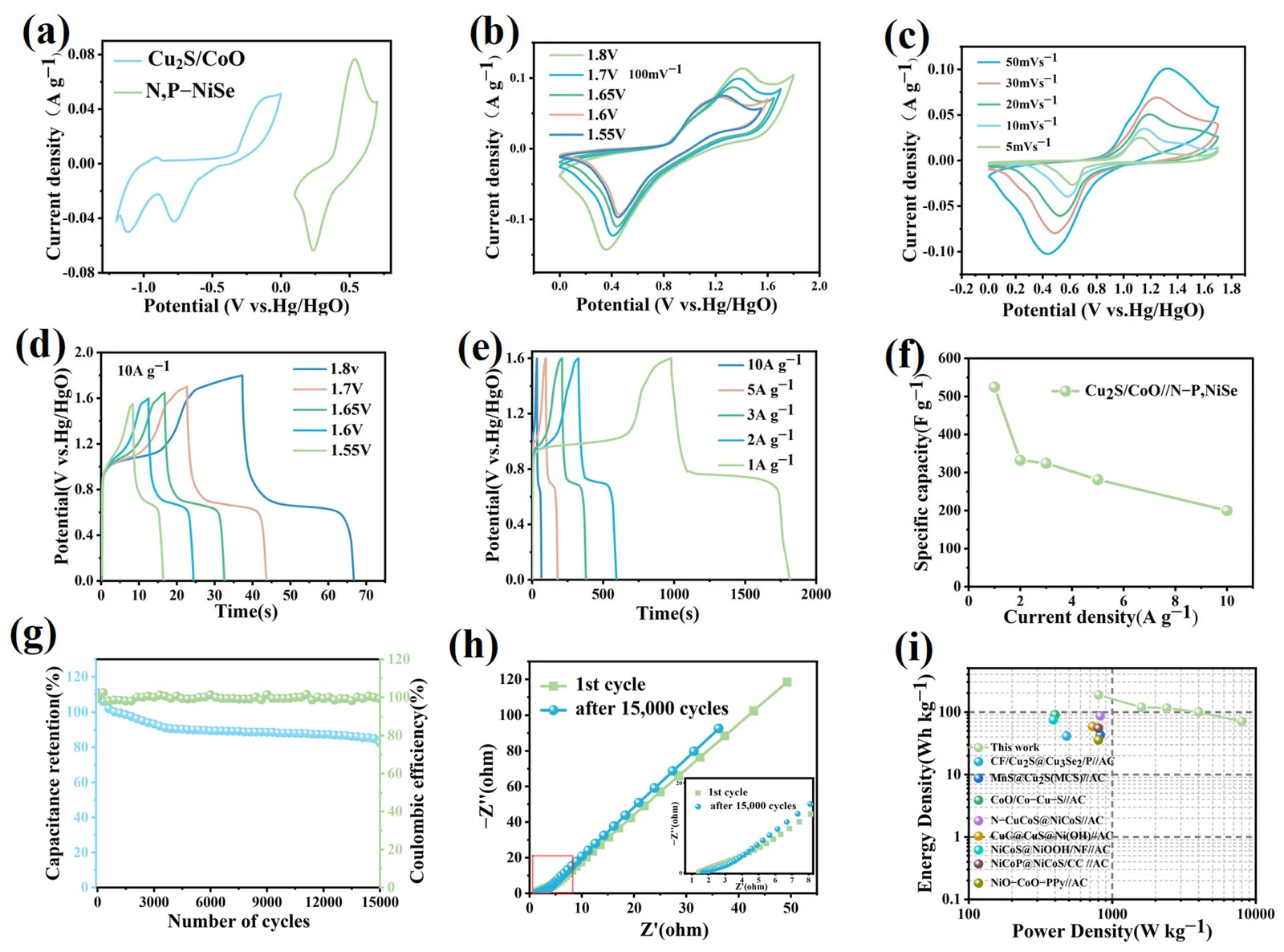
(**a**) CV curves of Cu_2_S/CoO and N, P-NiSe. (**b**,**c**) CV curves of the device at different operating voltages and scan rates. (**d**,**e**) GCD profiles under various voltages and current densities. (**f**) Specific capacitance of the device at different current densities. (**g**) Cycling stability and Coulombic efficiency. (**h**) Resistance comparison before and after 15,000 cycles. (**i**) Energy and power density plots of various devices.

To evaluate the practical device performance of Cu_2_S/CoO, an asymmetric supercapacitor (ASC) was assembled with N, P-NiSe as the positive electrode and Cu_2_S/CoO as the negative electrode, with 6 mol L^−1^ KOH used as the electrolyte (Figure 5a). Single-electrode tests determined the potential windows of N, P-NiSe and Cu_2_S/CoO to be 0–0.5 V and −1.2–0 V, respectively. CV measurements of the full device revealed severe polarization when the voltage window exceeded 1.8 V; accordingly, 1.7 V was selected as the optimal operating voltage (Figure 5b). The CV curves collected at scan rates of 5–50 mV s^−1^ within 0–1.7 V maintained consistent profiles, demonstrating stable ion and electron transport kinetics inside the electrodes (Figure 5c). GCD measurements were carried out over 0–1.6 V to avoid overly prolonged charging durations at low current densities, which would otherwise impair efficient charge–discharge cycling. The GCD curves under various potential windows display regular shapes (Figure 5d). At current densities of 1, 2, 3, 5, and 10 A g^−1^, the specific capacitances of the ASC calculated based on the total mass of active materials of both positive and negative electrodes were 524.4, 332.5, 324.4, 281.3, and 200 F g^−1^, respectively (Figure 5e,f). Long-cycle measurements show that the device retained 82% of its initial capacitance after 15,000 GCD cycles, accompanied by notably increased solution resistance after cycling (Figure 5g,h). Figure 5i presents the Ragone curve, revealing the mutual restriction between energy density and power density. At a current density of 1 A g^−1^, based on the total mass of active materials loaded on both positive and negative electrodes, the assembled device delivered an energy density of 186.45 Wh kg^−1^ at a power density of 800.02 W kg^−1^. When the power density increased to 7998.75 W kg^−1^ (corresponding to a current density of 10 A g^−1^), the energy density decreased to 71.1 Wh kg^−1^. This overall storage capability outperforms or is close to plenty of related devices published previously [28,46,47,48,49,50,51,52].

## 3. Materials and Methods

### 3.1. Materials

Cobalt nitrate hexahydrate (Co(NO_3_)_2_·6H_2_O), thiourea (CH_4_N_2_S), urea (CH_4_N_2_O), potassium hydroxide (KOH)) and absolute ethanol (Aladdin Reagent Co., Ltd., Shanghai, China). Ethylene glycol (C_2_H_6_O_2_) (Tianjin Damao Chemical Reagent Co., Ltd., Tianjin, China), ethylenediamine (C_2_H_8_N_2_) (Tianjin Damao Chemical Reagent Co., Ltd., Tianjin, China). Copper foam (1.5 mm thickness) (Kunshan Tanqianlang Electronic Materials Co., Ltd., Kunshan, China). Ultrapure water produced in-house was used throughout the experiments. All reagents were used as received without further purification.

### 3.2. Preparation of Cu_2_S/CoO Composite

**Figure 6:**
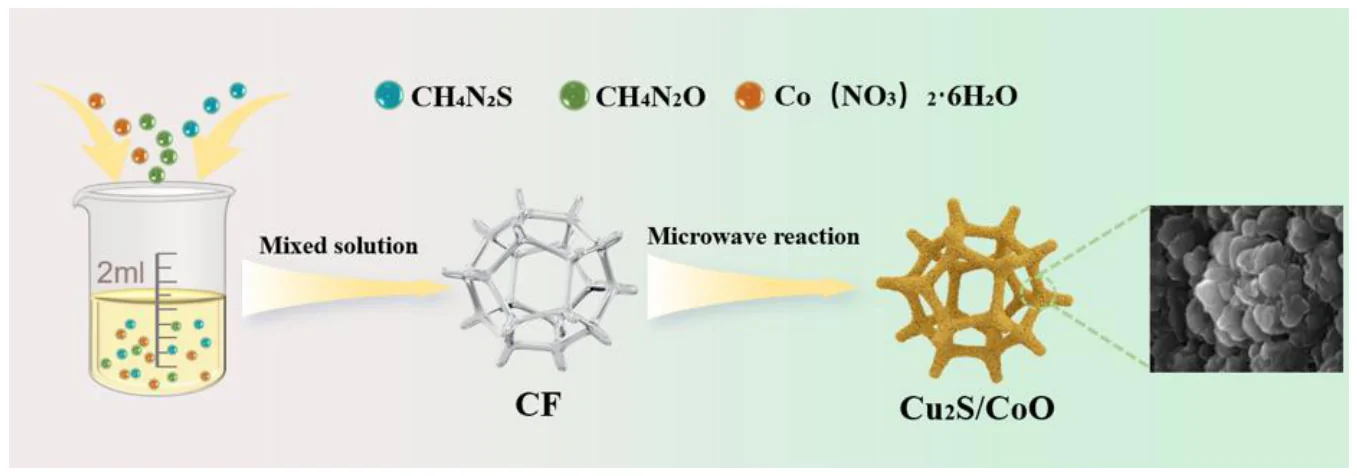
Schematic illustration of the fabrication process of the Cu_2_S/CoO composite.

First, copper foam pieces (1 cm × 1 cm) were cut and sequentially cleaned by ultrasonication in dilute hydrochloric acid, deionized water, and absolute ethanol. The cleaned foam was vacuum-dried at 80 °C for 12 h. Co(NO_3_)_2_·6H_2_O (10 mg), thiourea (10 mg, CH_4_N_2_S), urea (5 mg, CH_4_N_2_O), ethylene glycol (1 mL, C_2_H_6_O_2_), and ethylenediamine (0.8 mL, C_2_H_8_N_2_) were dissolved under magnetic stirring to form a pale yellow transparent solution. The pretreated copper foam was then immersed in the solution and heated at 60 °C for 10 min, followed by transfer to a 10 mL heat-resistant crucible for microwave treatment under the conditions specified in Table 2. The resulting sample was thoroughly rinsed with deionized water and absolute ethanol, and then vacuum-dried at 80 °C for 10 h. In this synthetic system, copper foam serves as both the substrate and copper source. The mass loading of the Cu_2_S/CoO composite was obtained from the weight difference of the copper foam before and after the microwave reaction. The detailed fabrication procedure is illustrated in Figure 6.

### 3.3. Material Characterizations

The crystalline structure of the samples was characterized by X-ray diffraction (XRD, D8 AVANCE, Bruker, Karlsruhe, Germany) with Cu Kα radiation. The surface elemental composition and chemical valence states were identified by X-ray photoelectron spectroscopy (XPS, Thermo Scientific K-Alpha, Thermo Scientific, Waltham, MA, USA). The morphology and microstructure were examined by scanning electron microscopy (SEM, MAIA 3LMH, TESCAN, Brno, Czech Republic) and transmission electron microscopy (TEM, JEOL JEM-F200, JEOL, Tokyo, Japan). High-resolution TEM (HRTEM) and selected area electron diffraction (SAED) were further employed to observe lattice fringes, measure interplanar spacing, and confirm the crystallinity of the products.

### 3.4. Electrochemical Measurements

Cyclic voltammetry (CV), galvanostatic charge–discharge (GCD), and electrochemical impedance spectroscopy (EIS) were conducted on a CHI660E electrochemical workstation (CH Instruments, Austin, TX, USA) in a three-electrode configuration. A platinum sheet, Hg/HgO, and 2 M KOH solution were used as the counter electrode, reference electrode, and electrolyte, respectively. The cycling stability of the electrodes was evaluated on a LAND battery testing system (CT2001A, LAND Electronics, Wuhan, China). The specific capacitance Cs was calculated using the following equation [53,54]:

(7) $$Cs\left({Fg}^{-1}\right)=\frac{I\times \Delta t}{m\times \Delta V}$$

where Cs is the specific capacitance, Δt (s) and ΔV (V) are the discharge time and potential window, respectively, I (A) is the applied current, and m (g) is the mass loading of the active materials. An asymmetric supercapacitor (ASC) was assembled using the as-fabricated sample as the negative electrode and previously reported N, P-co-doped NiSe as the positive electrode [55]. The mass ratio between the two electrodes was determined by the following equation [56,57]:

(8) $$\frac{m_{-}}{m_{+}}=\frac{C_{s+}\times {\Delta V}_{+}}{C_{s-}\times {\Delta V}_{-}}$$

The energy density (E) and power density (P) were calculated using the following equations [58]:

(9) $${E(Wh\;kg}^{-1})=\frac{C_{s}\times {\left(\Delta V\right)}^{2}}{7.2}$$

(10) $${P(W\;kg)}^{-1}=\frac{3600\times E}{\Delta t}$$

## 4. Conclusions

In this work, a self-supported Cu_2_S/CoO electrode grown on copper foam was successfully fabricated via a rapid microwave synthetic strategy. Its microstructure and elemental composition were systematically characterised by XRD, SEM, EDS, and XPS. The results confirm that uniform Cu_2_S/CoO nanoparticles were firmly grown on the copper foam surface. By optimizing microwave power and reaction time, the optimal synthesis condition was determined to be 300 W for 8 min. The optimized Cu_2_S/CoO electrode possessed a high specific capacitance of 2898 F g^−1^, maintaining 84.1% of its initial capacitance following 30,000 long-term cycles. Furthermore, an asymmetric supercapacitor (ASC) was constructed where Cu_2_S/CoO acts as the battery-type negative electrode and N, P-NiSe serves as the positive electrode. The optimized N, P-NiSe//Cu_2_S/CoO ASC reached a maximum energy density of 186.45 Wh kg^−1^ at a power density of 800.02 W kg^−1^ and held 82% capacitance retention after 15,000 cycles. It is verified that the microwave-synthesized Cu_2_S/CoO composite can be regarded as a prospective electrode material for high-performance supercapacitors and advanced energy storage systems.

## Figures and Tables

**Table 1:** Comparison of electrochemical performance between Cu_2_S/CoO and other materials.

Material	Method	Specific Capacity	Retention Rate	Ref.
CoO/Co-Cu-S	Calcination	2300 F g^−1^ (2 A g^−1^)	5000 cycles, 96.5%	[28]
NiCo_2_O_4_/CuS	Hydrothermal	2169 F g^−1^ (0.5A g^−1^)	7000 cycles, 123%	[42]
NiCo_2_O_4_@WO_3_/Cu_2_S	Hydrothermal	1932 F g^−1^ (1A g^−1^)	20,000 cycles, 96.7%	[43]
NiO-CoO	Hydrothermal	298 F g^−1^ (10A g^−1^)	5000 cycles, 94.30%	[44]
CuS (Mo@CuS)	Solvothermal	3504.2 F g^−1^ (2A g^−1^)	5000 cycles, 74.8%	[45]
Cu_2_S/CoO	Microwave	2898F g^−1^ (1A g^−1^)	30,000 cycles, 84.1%	This work

**Table 2:** Cu_2_S/CoO under various reaction conditions.

Sample	Microwave Watts/W	Microwave Duration/min	Duration/s Capacitance/ F g^−1^ (1Ag^−1^)	Mass Loading/mg
P1	600 W	5 min	1165	1.5
P2	400 W	5 min	1424	1.5
P3	300 W	5 min	1750	1.5
T1	300 W	8 min	2898	1.6
T2	300 W	10 min	1924	1.6

## Data Availability

The data presented in this study are available on request from the corresponding author.

## Supplementary Materials

The following supporting information can be downloaded at: https://www.mdpi.com/article/10.3390/molecules31203715/s1.
