# Supplementary material for: Microwave-Assisted Rapid Synthesis of Cu2S/CoO Heterostructured Composites for High-Performance Asymmetric Supercapacitors
Source: Molecules. 2026 Oct 16;31(20):3715. doi: 10.3390/molecules31203715 (PMC13707925; doi:10.3390/molecules31203715)
Supplement: Supplementary file 1 [file molecules-31-03715-s001.zip › molecules-4606202-supplementary.pdf]

Microwave-Assisted Rapid Synthesis of Cu_2S/CoO Heterostructured Composites for High-Performance Asymmetric Supercapacitors

Yunxia Zhao, Yu Yang, Qianwen Duan, Shuxin Gong, Bowen Du, Zhen Lu*, Feng Feng*

[1] School of Chemistry and Chemical Engineering, Shanxi Datong University, Datong 037009, China

* Correspondence: luzhen0313@aliyun.com (Z.L.); feng-feng64@263.net (F.F.)

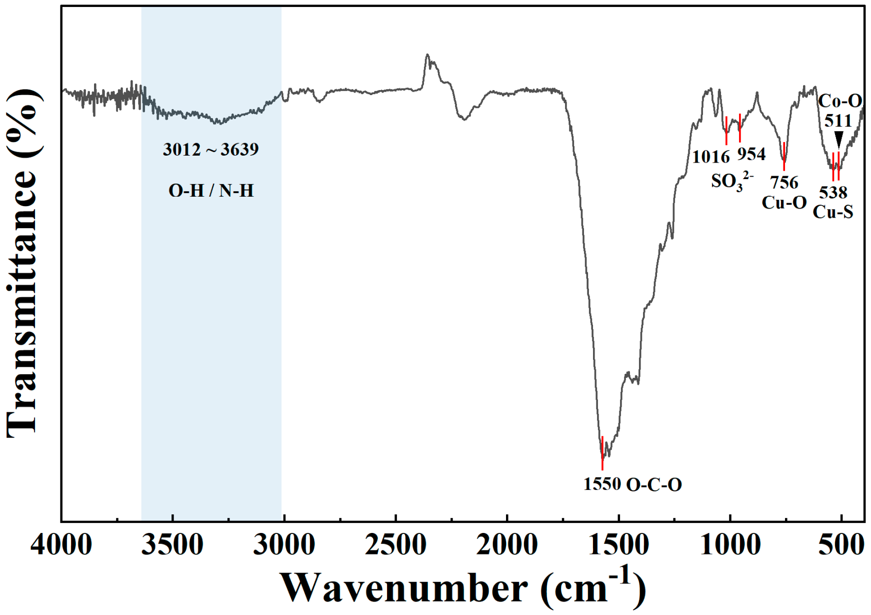

Figure S1. FTIR spectrum of the as-prepared Cu_2S/CoO sample.

FTIR characterization was performed on the Cu_2S/CoO sample. Obvious absorption peaks appear at 511, 538, 756, 954 and 1016 cm^{-1}. Among them, the absorption peak at 511 cm^{-1} is assigned to the stretching vibration of the Co–O bond, confirming the existence of the CoO phase in the sample. The absorption peak at 538 cm^{-1} corresponds to the stretching vibration of the Cu–S bond, verifying the Cu_2S component. During the formation of the Cu_2S/CoO heterostructure, electronic interaction and lattice mismatch occur at the interface, which alter the force constant of chemical bonds. The Cu–S peak exhibits a red shift and the Co–O peak shows a blue shift; these two peaks shift toward each other and finally merge into one single peak as displayed in the spectrum. The absorption band at 756 cm^{-1} is attributed to the stretching vibration of the Cu–O bond, indicating partial surface oxidation of Cu_2S or the presence of a small amount of copper oxide species. The peaks at 954 and 1016 cm^{-1} are jointly assigned to the S–O stretching vibration of sulfate SO_4^{2-}, demonstrating the residual sulfate species in the sample. The broad band at 1550 cm^{-1} (1535–1565 cm^{-1}) is assigned to the asymmetric stretching of O−C−O for surface-adsorbed bidentate carbonate on Cu_2S/CoO. The broad absorption band in the range of 3012–3639 cm^{-1} originates from O–H stretching vibration. The higher wavenumber component may derive from isolated surface hydroxyl groups, while the broad band at lower wavenumbers is related to hydrogen-bonded hydroxyl groups and physically adsorbed water.

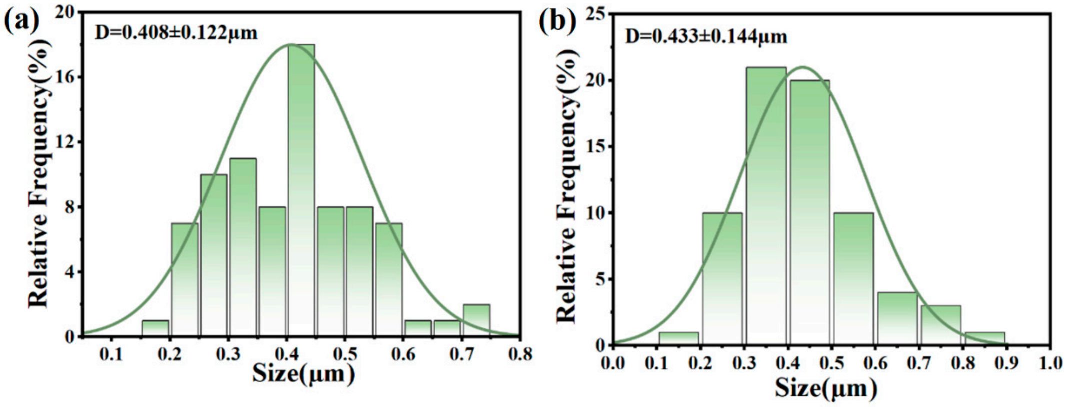

Figure S2. Particle size distribution histograms of Cu_2S/CoO synthesized at 300 W for (a) 8 min and (b)10 min, derived from SEM statistics.

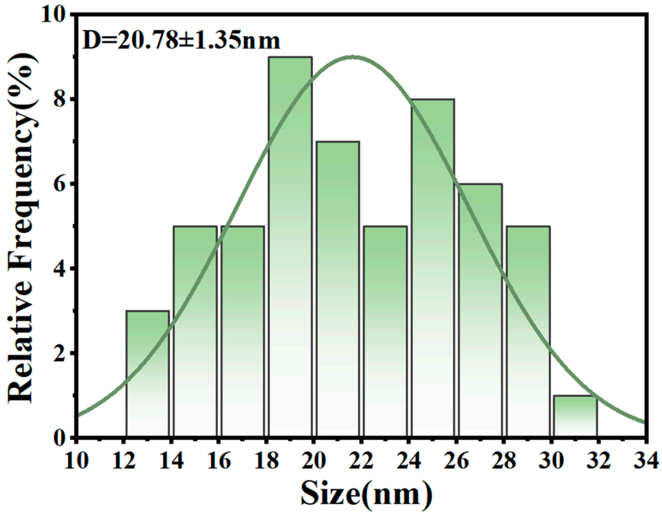

Figure S3. TEM grain size distribution histogram of the Cu_2S/CoO composite.

The porous structure and specific surface area of the Cu_2S/CoO heterostructure were characterized by nitrogen adsorption–desorption measurement. As displayed in Figure S4(a), the isotherm belongs to a typical Type IV curve with an obvious H3 hysteresis loop at high relative pressure P/P0 = 0.8-1.0, which indicates the presence of slit-like mesopores originating from the stacking of Cu_2S/CoO nanoparticles. No saturated adsorption plateau is observed, in good agreement with the H3-type hysteresis characteristic of aggregated particles. The BET specific surface area and total pore volume are summarized in Table S1. The BJH pore size distribution derived from the desorption branch (Figure S4(b)) reveals that the pores are mainly distributed in the mesopore range, with an average pore diameter of 26.42 nm. Such a mesoporous structure can shorten the ion diffusion distance, provide abundant accessible channels for electrolyte penetration, and expose more electroactive sites, which is favorable for the charge storage of the Cu_2S/CoO electrode.

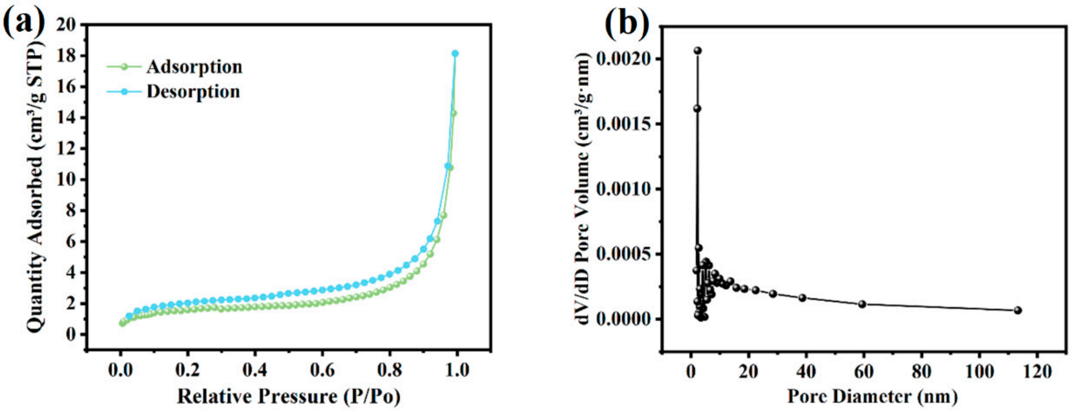

Figure S4. (a) Nitrogen adsorption–desorption isotherms and (b) BJH pore size distribution curve of the Cu_2S/CoO heterostructure.

Table S1. BET specific surface area, total pore volume, and average pore diameter of the Cu_2S/CoO heterostructure.

Sample	BET specific surface area (m^2 g^{-1})	Total pore volume (cm^3 g^{-1})	Average pore diameter (nm)
Cu_2S/CoO	6.0758	0.02762	26.4222

Table S2. Electrochemical impedance spectroscopy (EIS) fitting parameters for the as-prepared Cu2S/CoO electrode before cycling.

Element	Parameter Description	Value	Error (%)
Rs	Electrolyte solution resistance	0.671603 Ω	1.2239
CPE1-T	Constant phase element coefficient	0.0055609 $F\cdot sP^{-1}$	1.81
CPE1-P	Constant phase element exponent	0.73654	4.998
Rct	Charge-transfer resistance	0.99507 Ω	2.58
χ^2	Goodness of fit	0.00093529	1.1128

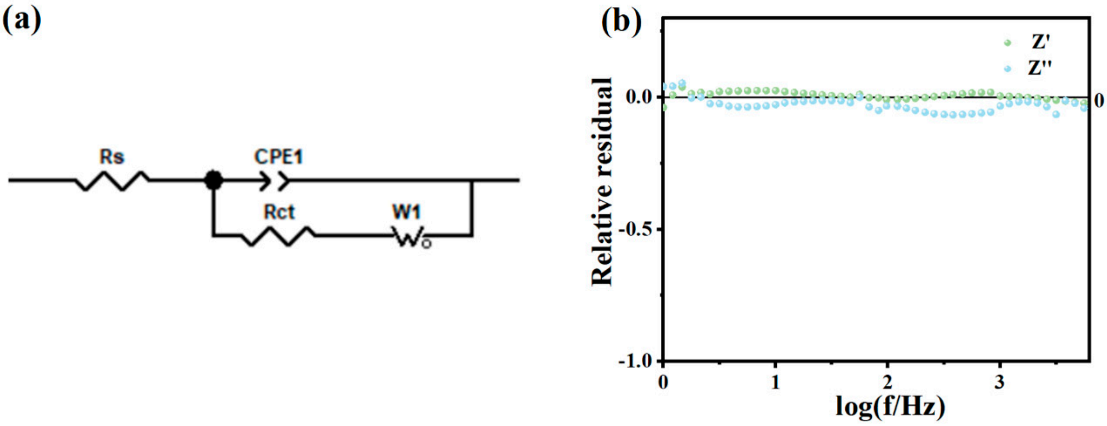

Figure S5. (a) Equivalent circuit for EIS fitting of Cu_2S/CoO electrode. (b) Relative residuals of Z′ and Z″ from EIS fitting.

Electrochemical impedance spectra were fitted using the equivalent circuit displayed in Figure S5(a). In this circuit, Rs represents the series resistance originating from the electrolyte and contact resistance. Rct corresponds to the charge-transfer resistance at the electrode–electrolyte interface. CPE1 is the constant phase element accounting for the non-ideal capacitive behavior of the electrode surface. Figure S5(b) presents the relative residuals of impedance real part Z' and imaginary part Z''. The residuals are distributed near zero over the whole frequency range, demonstrating that the proposed equivalent circuit can well describe the impedance response of the Cu_2S/CoO electrode.

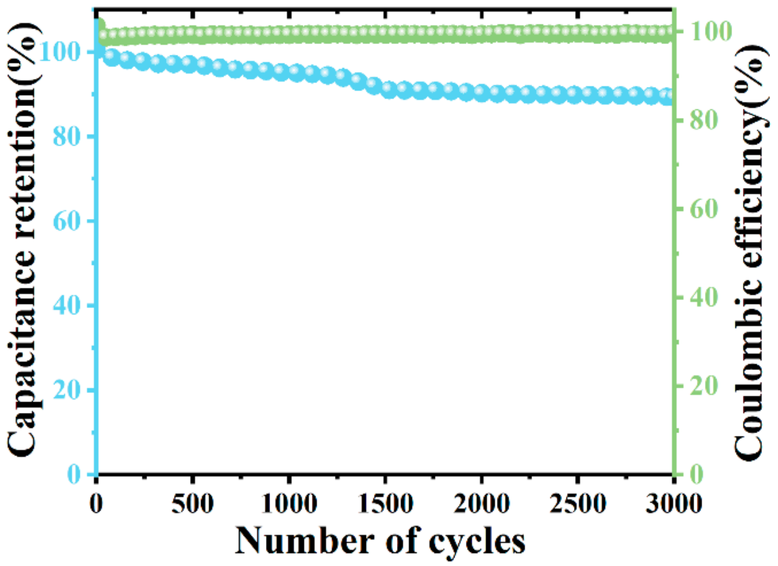

Figure S6. Long-term cycling stability of the Cu_2S/CoOelectrode at 1 A g^{-1} over 3000 cycles.

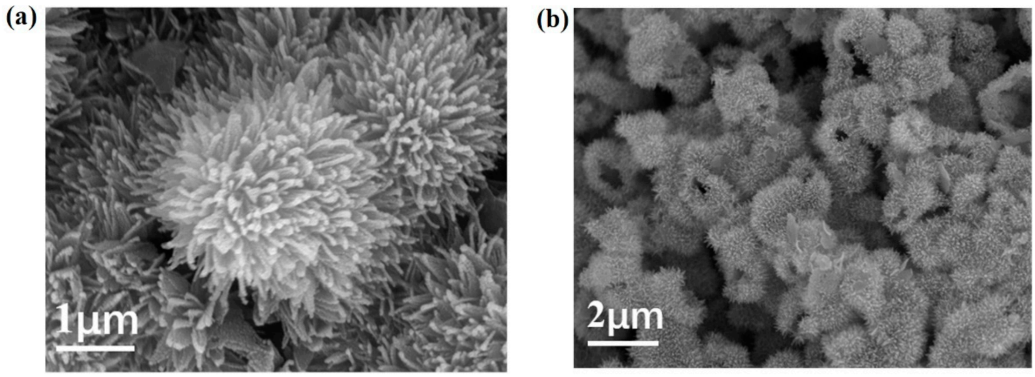

Figure S7. SEM image of Cu_2S/CoO electrode after 30000 long cycles.

Where $m^-$and m^+ are the mass of active materials on the negative and positive electrodes, respectively. $C_s^-$and C_s^+ denote the specific capacitances of anode and cathode, while $\Delta V_+$and ΔV-

correspond to their stable potential windows obtained by three-electrode measurements.In this work, the as-prepared Cu_2S/CoO was used as the anode, with C_s^-=2898Fg^{-1} and a potential window of 0–1.0 V. The N, P-co-doped NiSe cathode exhibits C_s^+=3184Fg^{-1} within the potential range of 0–0.5 V. According to Equation (8), the theoretical mass ratio m^-:$m^+$was calculated to be 0.55:1. The active mass loadings of the anode and cathode were 1.10 mg and 2.00 mg, respectively. Subsequently, the relevant parameters of the two-electrode device can be calculated according to Equations (9) and (10).

The equation $i(v)=k_1v+k_2v^{1/2}$ is used to quantitatively decouple the pseudocapacitive and diffusion-controlled contributions from CV curves measured at different scan rates. At a fixed potential V, the current i obtained under various scan rates v is substituted into the equation. Linear fitting is performed by plotting $i/v^{1/2}$ against $v^{1/2}$,where the intercept and slope of the fitted line correspond to k_1 and k_2, respectively. The current originating from pseudocapacitance at this potential is calculated as k_1v.The pseudocapacitive capacity is acquired by integrating the pseudocapacitive current curve over the entire potential window, while the total capacity is obtained from the integration of the original CV curve.The proportion of pseudocapacitive contribution equals pseudocapacitive capacity divided by total capacity, and the remaining fraction represents the diffusion-controlled contribution. This fitting assumes that the crystal structure, morphology and active sites of the electrode remain unchanged during tests at different scan rates. However, slight structural reconstruction and surface phase transformation may occur under high scan rates or after long cycles, violating this assumption and inducing fitting errors.
